# Assessing the impact of CD73 inhibition on overcoming anti-EGFR resistance in glioma cells

**DOI:** 10.32604/or.2024.055508

**Published:** 2025-03-19

**Authors:** LUIZ FERNANDO LOPES SILVA, JULIETE NATHALI SCHOLL, AUGUSTO FERREIRA WEBER, CAMILA KEHL DIAS, PAULINE RAFAELA PIZZATO, VINíCIUS PIERDONá LIMA, JEAN SÉVIGNY, ANA MARIA OLIVEIRA BATTASTINI, FABRÍCIO FIGUEIRÓ

**Affiliations:** 1 Programa de Pós-Graduação em Ciências Biológicas: Bioquímica, Instituto de Ciências Básicas da Saúde, Universidade Federal do Rio Grande do Sul, Porto Alegre, RS 90035-003, Brazil; 2 Departamento de Bioquímica, Instituto de Ciências Básicas da Saúde, Universidade Federal do Rio Grande do Sul, Porto Alegre, RS 90035-003, Brazil; 3 Département de microbiologie-infectiologie et d’immunologie, Faculté de Médecine, Université Laval, Québec City, G1V 0A6, Canada; 4 Axe maladies infectieuses et immunitaires, Centre de recherche du CHU de Québec, Université Laval, Québec City, G1V 4G2, Canada

**Keywords:** Glioblastoma (GB), Epidermal growth factor receptor (EGFR), CD73, Chemoresistance, Tyrphostin

## Abstract

**Objectives:**

Glioblastoma (GB) is a grade IV glial tumor characterized by high malignancy and dismal prognosis, primarily due to high recurrence rates and therapeutic resistance. The epidermal growth factor receptor (EGFR), a receptor tyrosine kinase (RTK), regulates signaling pathways, including cell growth, proliferation, survival, migration, and cell death. Many cancers utilize immune checkpoints (ICs) to attenuate immune responses. CD73 is an enzyme that functions as an IC by hydrolyzing AMP to adenosine, suppressing immune cells in the tumor microenvironment. However, the role of CD73 in resistance to EGFR inhibitors is poorly understood. This study aims to elucidate the resistance mechanisms induced by anti-EGFR treatment and to evaluate an anti-CD73 approach to overcome resistance mediated by anti-EGFR monotherapy.

**Methods:**

The U251 GB cell line was treated with AG1478, an EGFR inhibitor, and the resistance markers MRP-1, PD-L1, and CD73 were evaluated using flow cytometry. Additionally, we assessed the combination effects of AG1478 and APCP (an EGFR and a CD73 inhibitor, respectively) on cell cycle progression, proliferation, apoptosis, and migration *in vitro*.

**Results:**

We observed high EGFR, PD-L1, and CD73 expression in human GB cells. The treatment with AG1478 increased the expression of resistance markers MRP-1, PD-L1, and CD73, whereas it decreased CTLA-4. The combination of AG1478 and APCP did not alter proliferation or apoptosis but interfered with cell cycling, arresting the cells in the G1 phase, decreasing cell motility and partially reversing MRP-1 overexpression.

**Conclusion:**

In summary, our findings indicate that CD73 inhibition has a modest effect in overcoming resistance to EGFR monotherapy *in vitro*. Thus, further *in vivo* studies are needed, as the inhibition of both EGFR and CD73 affects cells in the tumor microenvironment and could potentially enhance anti-tumor immunity.

## Introduction

Glioblastoma (GB) is the most prevalent and aggressive primary tumor of the central nervous system (CNS), accounting for 50.9% of all malignant brain tumors [1]. The World Health Organization (WHO) classifies GB as a grade IV malignancy, underscoring its highly invasive nature and poor prognosis [2]. Standard treatment protocols for GB typically include maximal surgical resection followed by radio- and chemotherapy with temozolomide (TMZ) [3]. Notwithstanding, the median survival for GB patients remains less than 15 months post-diagnosis [3,4]. Over the years, the therapeutic landscape for GB has seen minimal evolution despite the numerous clinical trials [5,6] conducted since establishing the current treatment paradigm in 2005 [4]. The rapid progression of tumors, the blood-brain barrier, the neuroplasticity and regenerative capacity of treatment-resistant GB stem cells, and the intricate cellular and molecular heterogeneity within the tumor microenvironment are significant obstacles to current treatment approaches [7–9].

Genome instability and sustained proliferative signaling are GB hallmarks, driving its aggressive behavior [10]. Among the genetic alterations in GB, the epidermal growth factor receptor (EGFR) is one of the most mutated genes (approximately 50% of cases) [11]. These alterations typically manifest as gene amplification (40%) or overexpression (60%) [12]. The most prevalent mutation, EGFR variant III (EGFRvIII), is a constitutively active form of the receptor, which is associated with enhanced tumor aggressiveness and a worse prognosis for GB patients [13]. Given the prominence of EGFR and EGFRvIII in GB pathophysiology, these molecules have been primary targets for therapeutic intervention. In this context, the tyrosine kinase inhibitor, tyrphostin AG1478, has been reported to effectively impair EGFR autophosphorylation and signaling in experimental GB [14–16]. Unfortunately, despite their theoretical promise, clinical trials targeting EGFR in GB have yielded disappointing results, with only a small subset of patients deriving meaningful therapeutic benefits [17,18]. This limited response is often linked to resistance emergence throughout treatment.

EGFR inhibitors have shown promising results in preclinical studies combined with other therapeutic agents [16,19,20]. Combining EFGR inhibitors with other target therapies might be an interesting strategy to minimize the acquired resistance and improve clinical outcomes through synergistic inhibition of downstream growth pathways [21]. In light of this, a significant association between EGFR and CD73 signaling pathways has been observed in several tumors [22,23]. CD73, also known as ecto-5′-nucleotidase, is a cell membrane protein responsible for hydrolyzing AMP into adenosine (ADO), an immunosuppressive molecule. CD73 overexpression has been identified in several cancer cells and tumor biopsies and is associated with reduced disease-free survival in GB patients [24,25]. CD73 interacts with extracellular matrix components to promote adhesion, cell growth, epithelial-mesenchymal transition (EMT), invasion and to assist in pro-metastatic processes [26–28]. Furthermore, extracellular ADO production mediated by CD73 activity contributes to immunosuppression [29,30], cell proliferation [26], angiogenesis [31], and chemoresistance [32]. Efforts to target CD73 as a potential cancer therapy have primarily aimed at inhibiting its immunomodulatory activity to render immunologically “cold” tumors sensitive to checkpoint inhibitors [33–37].

Therefore, in light of CD73’s role in cancer cell survival and progression, it would be insightful to explore whether inhibiting CD73 in combination with the EGFR inhibitor tyrphostin AG1478 could enhance therapeutic responses in a GB *in vitro* model. Thus, this study aimed to explore the potential of an anti-CD73 approach to overcome resistance mediated by anti-EGFR monotherapy.

## Materials and Methods

### Chemicals

Dulbecco’s modified Eagle’s medium (DMEM, cat#12100046), Fetal Bovine Serum (FBS, cat#26140079), Amphotericin B (cat#15290026), penicillin/streptomycin (cat#15140148), and trypsin-EDTA (cat#15400054) solution were acquired from Gibco (Gibco BRL, Grand Island, NY, USA). Dimethylsulfoxide (DMSO, cat#D2438), Tyrphostin AG1478 (AG1478, cat#T4182), adenosine 5′-(α, β-methylene) diphosphate (APCP, cat# M3763), temozolomide (TMZ, cat#T2577) and Trypan Blue (cat# T8154) were obtained from Sigma-Aldrich (Burlington, MA, USA). AnnexinV-APC and PI, Active Caspase-3, anti-human EGFR, anti-human MRP-1, anti-human PD-L1, anti-CTLA-4, anti-human CD73 were purchased from BD Biosciences (BD Bioscience, Franklin Lakes, NJ, USA).

### GB cell culture

The U251-MG, U87-MG, U138-MG, T98G, and A172 human GB cell lines were acquired from American Type Culture Collection (ATCC, Rockville, MD, USA). Cells were cultured and maintained in DMEM containing 10% FBS, 0.5 U/mL penicillin/streptomycin and 100 g/mL Amphotericin B, under humidified conditions (37°C, 5% CO_2_), for up to 30 passages. The cell lines were routinely monitored for *Mycoplasma sp*. contamination by PCR, following the method described by Fan et al. [38] and using the following primer sequences: F: ACACCATGGGAGCTGGTAAT; R: CGTAGGTTGTACTCCGTAGAAAGG.

### Cell viability assay

The U251-MG cell line was seeded in 24-well plates (1.1 × 10^4^ cells/well). When semi-confluence was reached, cells were treated with AG1478 (EGFR inhibitor, Sigma-Aldrich, Burlington, MA, USA) in concentrations of 5 to 80 µM for 48 h, under standard cell culture conditions (37°C in 5% CO_2_). Following 48 h of treatment, cells were washed twice with 1 × Phosphate Buffered Saline (1 × PBS, pH 7.4) and detached with 0.5% trypsin-EDTA solution. Then, cells were stained with Trypan Blue (0.1%) and immediately counted in a hemocytometer. In experiments investigating the role of the purinergic pathway, cells were plated and treated for 48 h with 35 μM AG1478 and/or 100 μM APCP (CD73 inhibitor, Sigma-Aldrich, Burlington, MA, USA) under standard cell culture conditions (37°C in 5% CO_2_). In TMZ experiments, the U251-MG were plated and treated for 48 h with 75, 250, and 500 µM of TMZ alone or in cotreatment with 35 μM AG1478.

### Hemolysis assay

The hemolytic capability of AG1478 was performed as previously described [39]. For this, peripheral blood samples of healthy donors (4 mL) were collected after a signed consent. The blood was centrifugated at 2500 g for 10 min (25°C, Eppendorf® Centrifuge 5804-R), and the erythrocytes were separated and washed three times with 1 × PBS (pH 7.0). Red blood cells were plated in 96-well plates (5 × 10^5^ cells/well) and treated with 10–250 μM AG1478 for 48 h, under usual cell culture conditions (37°C, 5% CO_2_). All controls necessary for the experiment were used (negative control, vehicle control, and positive control-Triton X-100 0.1%, Sigma-Aldrich, Burlington, MA, USA). After the end of treatment, the plate was centrifuged at 2250× *g* per 10 min (25°C, Eppendorf® Centrifuge 5804-R) and the hemoglobin detected into the culture supernatant was quantified using absorbance at 540 nm (SpectraMax® M5, Molecular Devices). The degree of hemolysis was calculated using the hemolytic ratio. The data acquired from the samples treated with AG1478 (ODtest) were normalized to positive (Triton X-100 0.1%, ODpos) and negative (untreated, ODneg) control samples to provide the hemolytic ratio (HR), as defined by the formula (1):

(1) $$HR\;\%=\frac{ODtest-ODneg}{ODpos-ODneg}\;\times \;100$$

### Assessment of EGFR protein expression in GB cell lines

All flow cytometry experiments were conducted as previously reported by our group [40]. We assessed EGFR expression, in untreated samples, of the U251-MG, U87-MG, U138-MG, T98G, and A172 human GB cell lines. Briefly, cells were cultured in 24-well plates (1.1 × 10^4^ cells/well), under typical cell culture conditions (37°C, 5% CO_2_), until they reached semi-confluence. Next, cells were washed with 1 × PBS (pH 7.4), trypsinized, and centrifuged twice at 400 × g for 6 min at 25°C. Following centrifugation, the cells were stained with anti-human EGFR (1:30, clone EGFR.1, cat#555997, BD Biosciences, Franklin Lakes, NJ, USA) for 30 min in the dark, on ice, and washed twice. Data acquisition was performed using flow cytometry (FACSCalibur, BD Biosciences, USA), and the results were assessed with FlowJo® v10.9.0 software (BD Biosciences, Franklin Lakes, NJ, USA). The most suitable GB cell line was chosen for subsequent experiments based on EGFR expression levels (U251-MG).

### Protein expression by flow cytometry

For the protein expression assessment, U251-MG was seeded in 24-well plates (1.1 × 10^4^ cells/well) and grown until semi-confluence, under standard cell culture conditions (37°C, 5% CO_2_). After, U251-MG was treated with AG1478 (35 µM) and/or 100 μM APCP for 48 h, also in standard *in vitro* culture settings (37°C, 5% CO_2_). At the end of treatments, cells were washed with blocking buffer (1 × PBS + 2% FBS) and stained with anti-human ecto-5′-nucleotidase/CD73 (1:200, cat. h5′NT-1L (I4,I5), J. Sevigny’s research lab, Canada, http://ectonucleotidases-ab.com, accessed on 18 November 2024 [41], FITC-conjugated goat anti-rabbit IgG secondary Ab (1:100, cat. 65-6111, Invitrogen, Austin, TX, USA), anti-human MRP-1 (1:50, clone QCRL-3, cat#557594), goat anti-mouse Alexa Fluor™ 488 (1:100, ThermoFisher, cat#A-11001), anti-Ki-67 (clone SolA15, 1:200; eBioscience, Cat# 11-5698-82), anti-human EGFR (1:30, clone EGFR.1, cat#555997), anti-human PD-L1 (1:100, clone MIH1, cat#558017), anti-CTLA-4 (1:10, clone BNI3, cat#560938) and/or anti-human CD73 (1:30, clone AD2, cat#550257) for 30 min on ice. Data were collected using flow cytometry (BD Accuri™ or FACSCalibur, BD Biosciences, USA) and analyzed with FlowJo® v10.9.0 software (BD Biosciences, Franklin Lakes, NJ, USA). A secondary antibody was used as a non-specific binding control (FITC-conjugated goat anti-rabbit IgG secondary Ab (1:100, cat. 65-6111, Invitrogen, USA).

### Annexin V-APC/PI and cell cycle

To evaluate cell death and cell cycle, 1.3 × 10^4^ cells/well of U251-MG cells were cultured in 24-well plates. After 24 h, cells were treated with IC_50_ values of AG1478 (35 μM) and/or 100 μM APCP for 48 h, under standard cell culture conditions (37°C, 5% CO_2_). Apoptotic and/or necrotic cells were quantified with Annexin V APC/Propidium Iodide (PI) double staining kit following the manufacturer’s guidelines (BD Biosciences, Franklin Lakes, NJ, USA). The U251-MG cells (10^5^ cells/mL) were suspended in a buffer comprising Annexin V-APC and PI for 15 min (BD Biosciences, San Diego, CA, USA, cat#550474). For cell cycle analysis, U251-MG cells (10^6^ cells/mL) were suspended in 300 µL staining solution containing Tris-HCl (0.5 mM, pH 7.6), trisodium citrate (3.5 mM), Nonidet P 40 (0.1% v/v), RNase (100 µg/mL, cat#R6513, Sigma-Aldrich, Burlington, MA, USA) and PI (50 µg/mL, cat#P4170 Sigma-Aldrich, Burlington, Massachusetts, USA), for 15 min in the dark. Data were acquired by a flow cytometrer (BD Accuri™ or FACSCalibur, BD Biosciences, USA), and analyzed using FlowJo® v10.9.0 software (BD Biosciences, Franklin Lakes, NJ, USA). For apoptotic cell death, cells were classified as follows: viable cells (Annexin V and PI negative), early apoptotic cells (Annexin V positive and PI negative), and late apoptotic or necrotic cells (Annexin V and PI positive or Annexin V negative and PI positive).

### Chemoresistance protein functional assay—CFDA

ThermoFisher® CFDA AM (5-Carboxyfluorescein Diacetate, Acetoxymethyl Ester, cat#C1354, ThermoFisher®, Franklin, MA, USA) probe was used to assess the capacity of some transmembrane transporters, which can be used by neoplastic cells as efflux pumps for antineoplastic drugs [42]. In the experiment that investigated the acute effect of treatment under efflux pumps, shown in Fig. 4C, cells were treated with IC_50_ values (35 µM) of AG1478 only in the CFDA incubation period, at the influx time. In the experiment that investigated the chronic effect of co-treatment, U251-MG cells were cultured in 6-well plates (8 × 10^4^ cells/well) and, after 24 h, were treated with IC_25_ values of AG1478 and 100 µM APCP for 48 h. Next, the cells were trypsinized and washed with 1 × PBS to initiate the chemoresistance functional assay, shown in Fig. 4D. To measure influx, cells were incubated with the probe (2 µM) in medium without FBS for 30 min. For the efflux, cells were incubated in media without FBS or CFDA for 90 min. During the influx and efflux, aliquots were taken to detect CFDA probe fluorescence and, consequently, the ability of the cells to extrude the probe by flow cytometry, using the cytometer BD Accuri C6™.

### Active caspase-3 immunocontent

We analyzed the immunocontent of active caspase-3 per the manufacturer’s instruction (PE Active Caspase-3 kit, 1:30, cat#550914, BD Biosciences, USA). In summary, U251-MG (10^6^ cells/mL) were incubated in BD Cytofix/Cytoperm™ solution (20 min on ice). Following this step, cells were washed with BD Perm/Wash™ buffer (1×) and stained with active caspase-3 antibody (PE Active Caspase-3 kit, cat#550914, BD Biosciences, USA) for 30 min at room temperature (RT) and then analyzed by the flow cytometer FACSCalibur (BD Biosciences, Franklin Lakes, NJ, USA). The results were assessed using FlowJo® v10.9.0 software (BD Biosciences, Franklin Lakes, NJ, USA).

### Wound healing assay

To assess the indication of cell migration, we performed the *in vitro* scratch assay following standard methods with modifications [43]. In brief, U251-MG were cultured in a 6-well plate (7 × 10^4^ cells/well) for 24 h under standard cell culture conditions (37°C, 5% CO_2_). A straight-edged and cell-free zone across the monolayer was established by employing a 200 µL sterile plastic micropipette tip to create a cross-wound in the bottom of the well. Next, the medium was replaced, containing AG1478 (35 μM) and/or 100 μM APCP and cells were incubated for 48 h in typical *in vitro* conditions (37°C, 5% CO_2_). Wound healing was analyzed by the Nikon Eclipse TE300 phase contrast microscopy (Melville, NY, USA) and images of each well were acquired at 0, 24, and 48 h with a Nikon Digital Camera DXM1200C (Düsseldorf, Germany). The scratch closure rate was analyzed by digitally drawing lines to represent the position of the migrating cells at the wounded edges with ImageJ® software (NIH, Bethesda, MD, USA).

### Transwell migration assay

To assess the migratory capacity of U251-MG cells, we performed the transwell migration assay following standard methods, with modifications [44]. The cell migration was evaluated using 24-well plates with Transwell inserts (8-μm pore size, cat#353097, Falcon®). Briefly, U251-MG cells (5 × 10^4^) were resuspended in 200 μL serum-free DMEM and added into the upper chambers. A total volume of 600 µL DMEM supplemented with 10% FBS was added to the lower chamber. The treatments were added in the suspension of U251MG cells at the concentration of 100 μM for APCP, 35 μM for AG1478, and co-treatment with APCP+AG1478 was also added. Following 48 h of treatment, the inserts were removed and cells remaining on the upper surface of the inserts were carefully removed using a cotton swab. Cells that spread to the underside surface were fixed with 20% methanol (cat#67-56-1, LiChrosolv®, Merck, Burlington, Massachusetts, USA) for 15 min (RT) and stained with 1% crystal violet for 20 min. Following the staining, five fields were randomly chosen and photographed using a microscope (EVOS XL Core system, Invitrogen™, Oslo-Norway) for cell counting.

### Statistical analysis

The Shapiro-Wilk test was carried out to verify multivariate normality. Kruskal-Wallis, a non-parametric test, was performed for data displaying non-normal distribution. Conversely, for data with normal distribution, a parametric test (one-way analysis of variance-ANOVA), followed by Tukey post-test was performed. All statistical data were carried out GraphPad Prism v8.0 software® (San Diego CA, USA). Data were expressed as the mean ± SD of at least three independent experiments. Statistical significance was determined as *p* < 0.05.

## Results

### AG1478 reduces glioblastoma cell viability without affecting healthy cell viability

**Figure 1:**
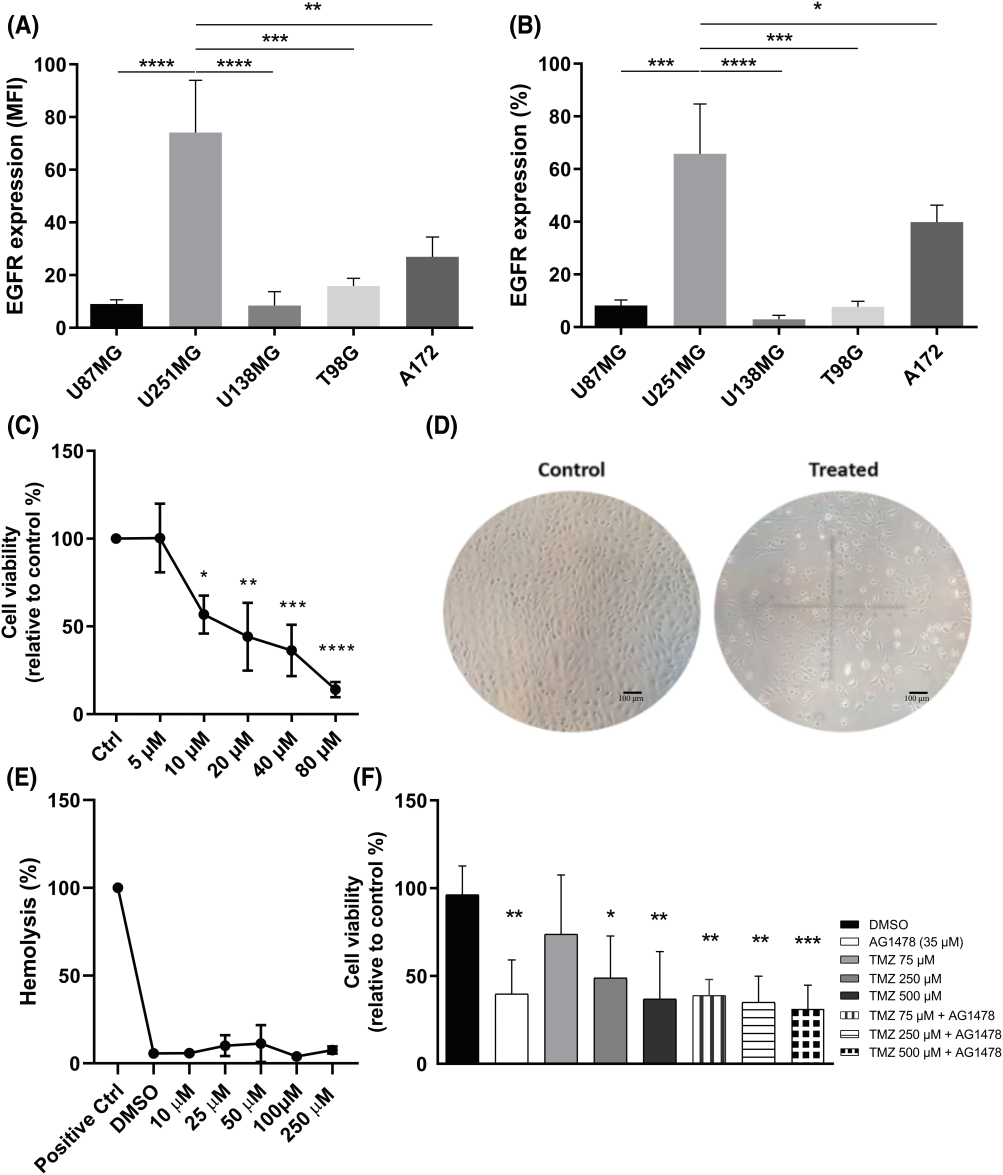
EGFR expression in human glioblastoma lines. (A) median fluorescence intensity (MFI) (B) percentage of cells expressing EGFR. Expression analysis was performed by flow cytometry. (C) Viable U251-MG cells exposed to treatment with Tyrphostin AG1478 for 48 h. (D) Cell morphology of U251-MG cells in the control group (Scale bar = 100 µm) (left) and in the AG1478-treated cells (right). Image obtained by microscopy. (E) Hemolysis of red blood cells treated with Tyrphostin AG1478 for 48 h. (F) U251-MG cells treated with Tyrphostin AG1478 and Temozolomide for 48 h. Cell viability analyses were performed by counting with Trypan Blue. *p < 0.05; **p < 0.01: ***p < 0.001; ****p < 0.0001.

First, EGFR expression was analyzed by flow cytometry in different human GB cell lines. U251-MG cells exhibited more EGFR-positive cells and a higher EGFR expression level than the other cell lines (Fig. 1A,B). Based on cell viability assay, 48 h of AG1478 treatment resulted in an IC_50_ value of 35 μM (Fig. 1C), accompanied by morphological changes indicative of cell death (Fig. 1D). To analyze potential cytotoxicity to healthy cells, a dose curve of AG1478 was carried out in the presence of red blood cells. There was no increase in hemolysis at any drug concentrations compared to the vehicle group (Fig. 1E). Also, regardless of concentration, the combination of AG1478 with TMZ has no synergistic effects on cell viability (Fig. 1F).

### Expression of CD73, PD-L1 and EGFR in U251MG

**Figure 2:**
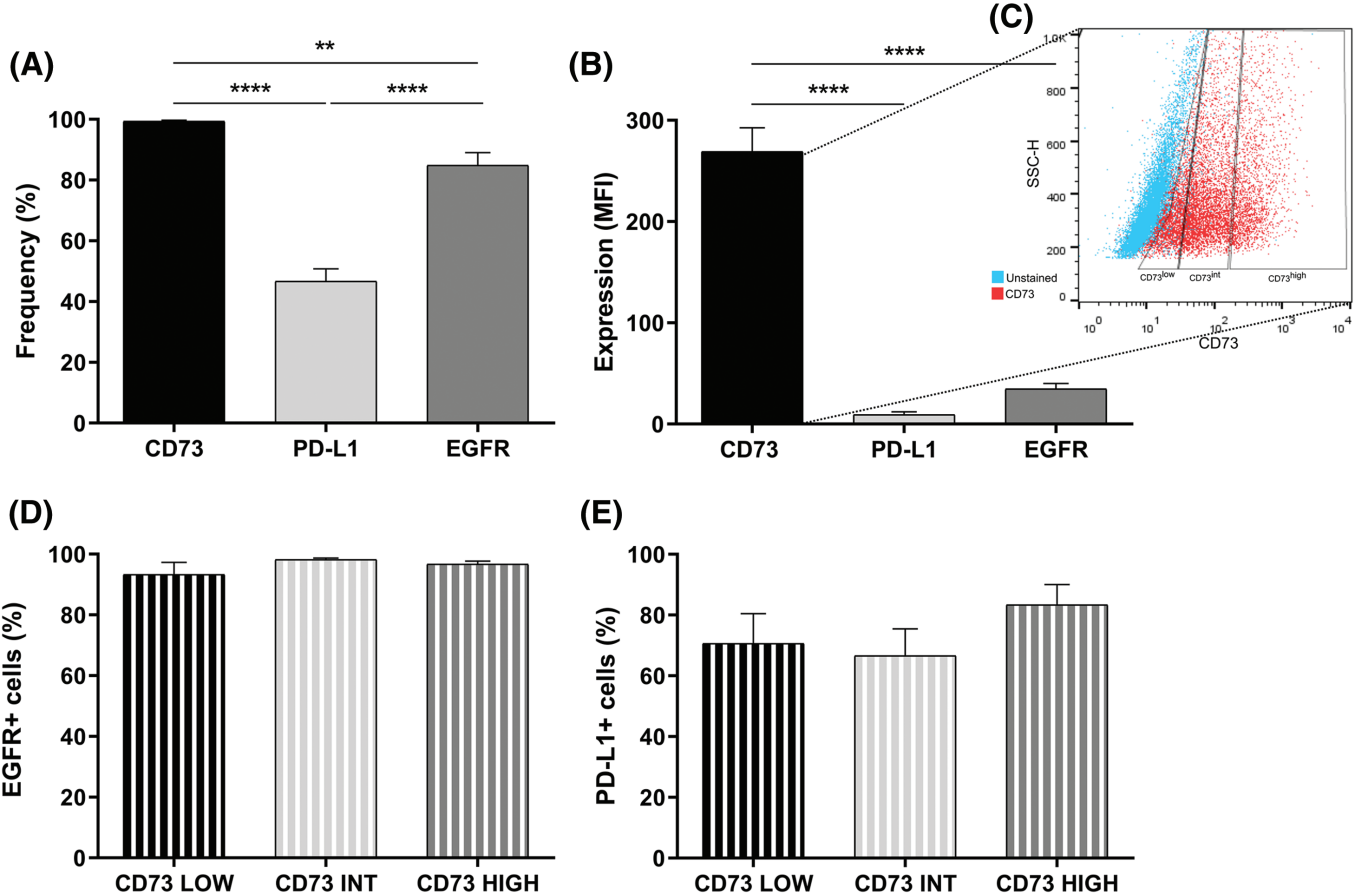
CD73, PD-L1, and EGFR expression in U251-MG cells. (A) Percentage and (B) median fluorescence intensity (MFI) of cells expressing CD73, PD-L1, and EGFR. (C) Subdivision of U251-MG cells in low, intermediate, and high CD73 expression. (D) EGFR expression in U251-MG cells with low (20%), intermediate (50%) or high expression of CD73 (20%) (E) Expression of PD-L1 in U251MG cells with low (20%), intermediate (50%) or high expression of CD73 (20%). Data determined by flow cytometry, n = 3. Data are displayed as means ± SD (**p < 0.01, ****p < 0.0001).

The expressions of essential proteins for glioma progression, CD73, PD-L1, and EGFR in U251MG cells were also evaluated. There was a high percentage of cells expressing CD73 and EGFR markers, and around 50% of cells expressing PD-L1 (Fig. 2A). Regarding the fluorescence intensity, GB cells overexpress CD73 compared to EGFR and PD-L1 (Fig. 2B). Afterwards, the total number of CD73-positive U251MG cells was divided into low expression of CD73 (up to 20% of CD73^+^ cells), intermediate expression of CD73 (the middle 50% of CD73^+^ cells) and high expression of CD73 (20% of CD73^+^ overexpressing cells) (Fig. 2C). EGFR and PD-L1 expression was not different in these three distinct subsets of CD73^+^ cells (Fig. 2D,E).

### Effect of anti-EGFR treatment on immune checkpoints and resistance

**Figure 3:**
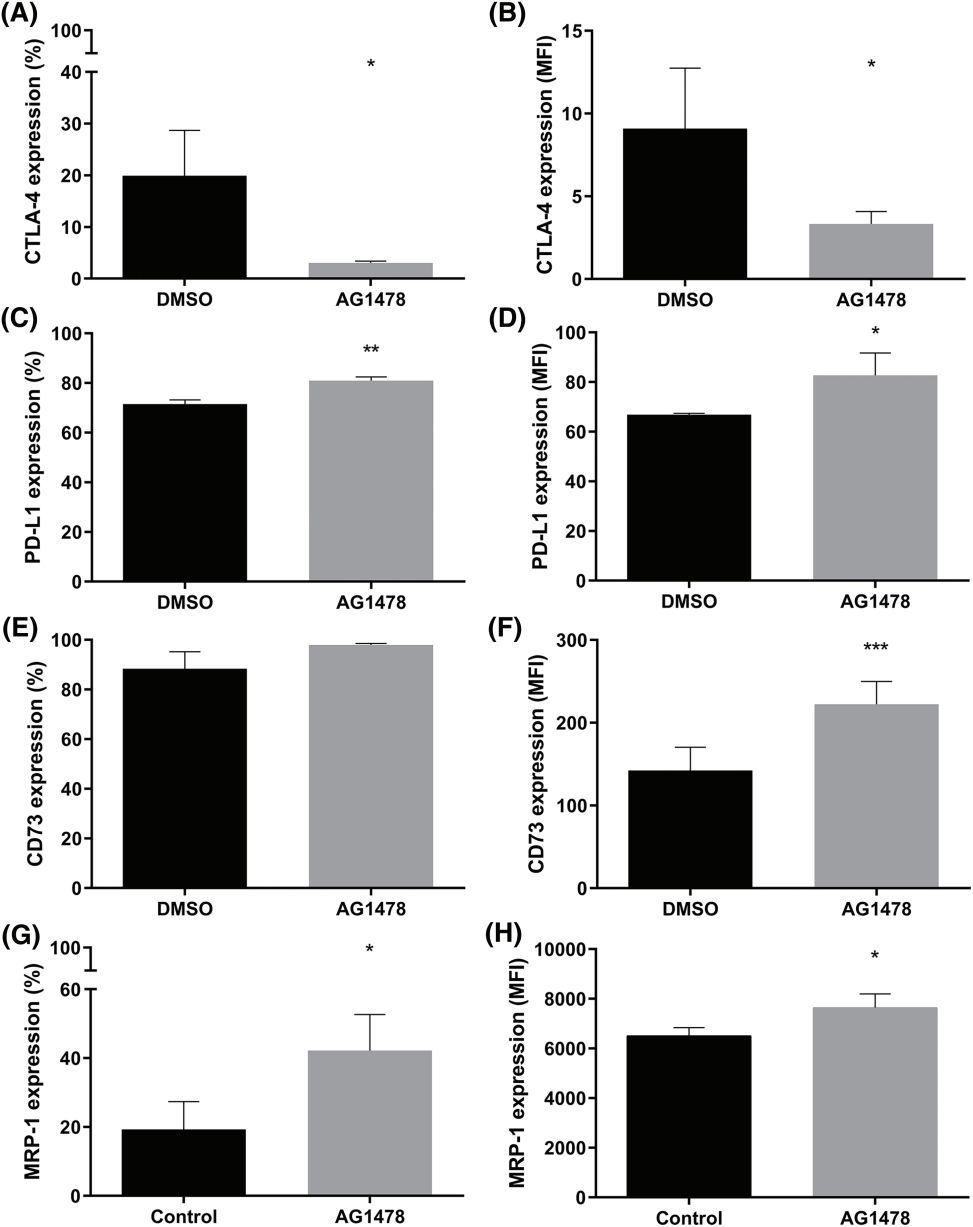
Resistance markers in the EGFR inhibitor AG1478-treated GB cells. (A) Percentage and (B) median fluorescence intensity (MFI) of cells expressing CTLA-4 treated with AG1478. (C) Percentage and (D) MFI of cells expressing PD-L1 treated with AG1478. (E) Percentage and (F) MFI of cells expressing CD73 treated with AG1478. (G) Percentage and (H) MFI of MRP-1 expressing cells treated with AG1478. Data obtained by flow cytometry, n = 3. Data are shown as means ± SD (*p < 0.05, **p < 0.01, ***p < 0.001).

To evaluate the effect of the EGFR inhibitor AG1478 in inducing resistance through immunological and tumoral pathways, the markers CTLA-4, PD-L1, CD73, and MRP-1 were assessed. AG1478 partially decreased CTLA-4 expression (Fig. 3A,B). However, the treatment increased PD-L1, CD73, and MRP-1 protein expression (Fig. 3C–H).

### The influence of EGFR inhibition on the purinergic pathway

**Figure 4:**
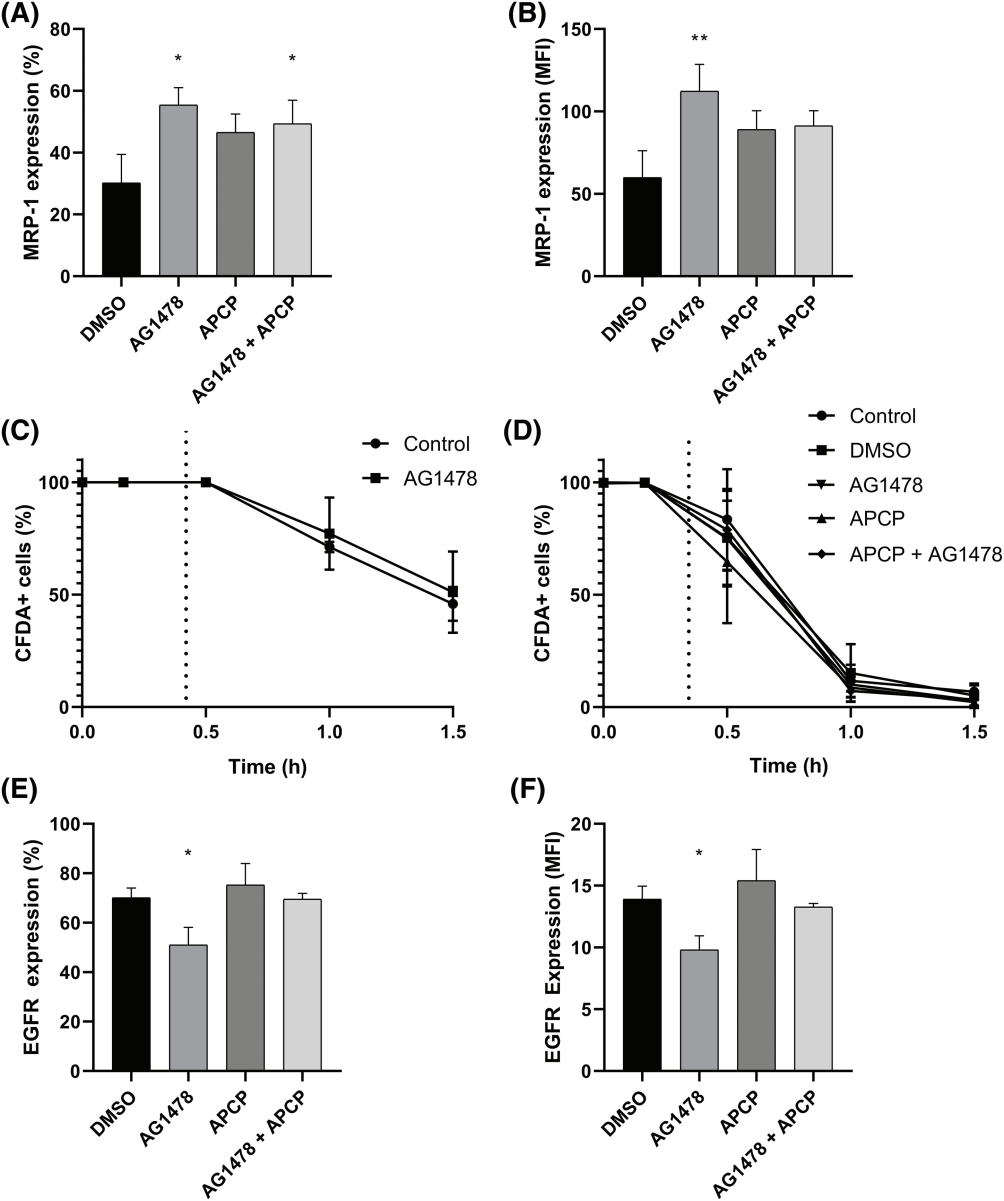
Cell viability and expression of MRP-1 and EGFR U251MG cells treated with Tyrphostin AG1478 associated or not with APCP for 48 h. (A) Percentage and (B) median fluorescence intensity (MFI) of cells expressing MRP-1 compared to control DMSO. (C) Activity of efflux drugs from cells treated with AG1478 acutely and (D) chronically—after 48 h as determined by flow cytometry. (E) percentage and (F) Median fluorescence intensity (MFI) of cells expressing from cells expressing EGFR compared to control DMSO. Data was collected by flow cytometry. Data are shown as mean ± SD (*p < 0.05, **p < 0.01).

To further explore the interaction between EGFR and CD73 inhibition, we sought to analyze the effects of these combined treatments on the expression of EGFR and MRP-1. The percentage of cells expressing MRP-1 was not significantly changed when comparing AG1478 and AG1478+the CD73 inhibitor APCP (Fig. 4A). On the other hand, APCP partially reversed the upregulation on MRP1 induced by AG1478 (Fig. 4B). However, both acute exposure and 48-h treatment with AG1478 did not change the functional activity of drug efflux pumps (Fig. 4C), even in the presence of APCP (Fig. 4D). In addition, AG1478 was able to reduce EGFR expression, apart from its well-known antagonistic effect (Fig. 4E). Interestingly, EGFR expression after combined treatment was reverted to control parameters (Fig. 4F).

### The cell death characteristics after co-inhibition of EGFR and CD73

**Figure 5:**
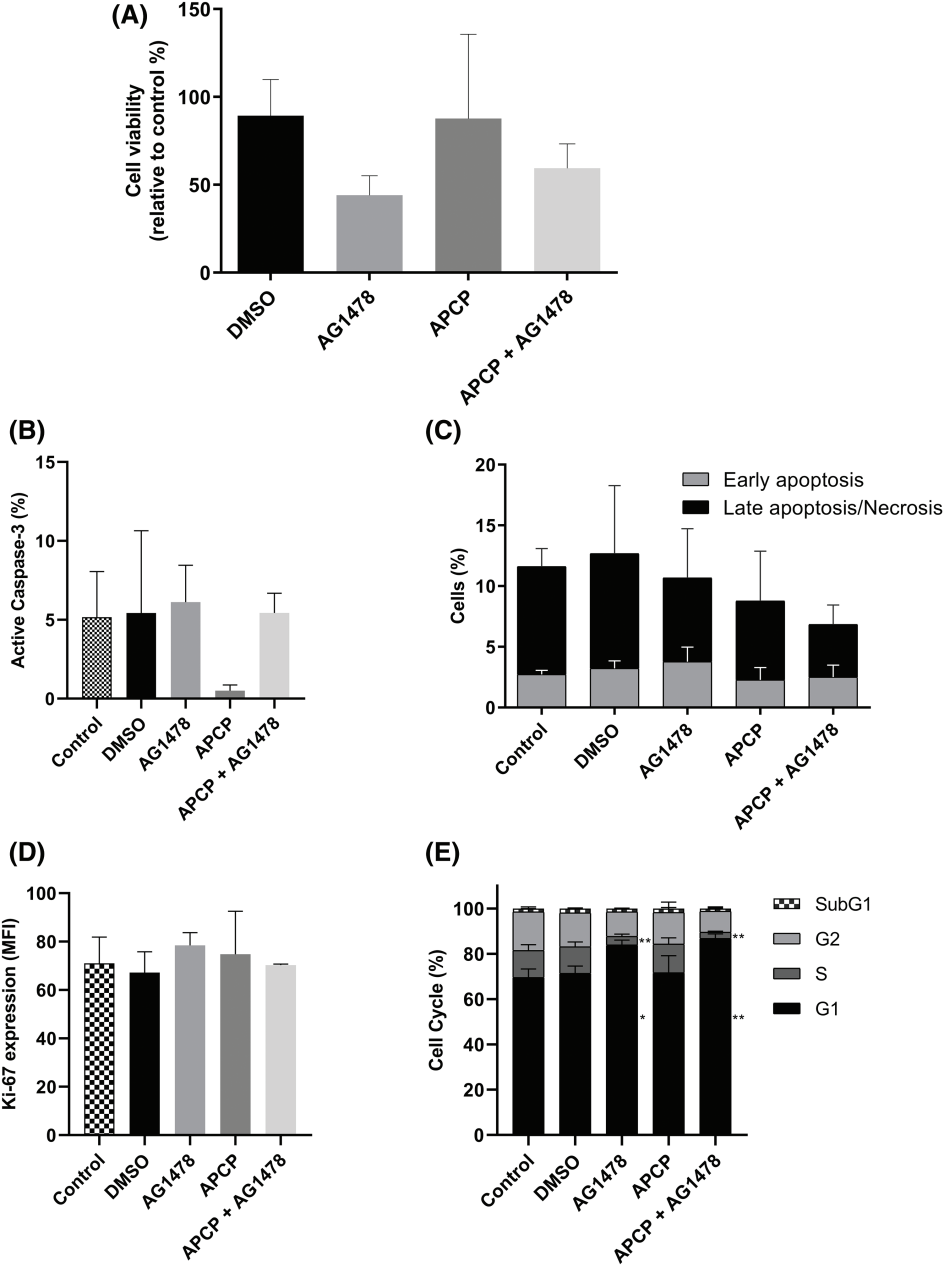
Cell death of GB cells after treatments with APCP and AG1478. (A) Viability of cells treated with AG1478 and APCP combined or not. Data determined by Trypan blue exclusion count. (B) Percentage of cells expressing active caspase-3. (C) Cells undergoing apoptosis or necrosis as determined by flow cytometry. (D) Expression of Ki-67 by median fluorescence intensity (MFI). (E) Percentage of cells in each cell cycle stage. Data are shown as means ± SD (*p < 0.05, **p < 0.01).

Next, we shifted to the perspective of resistance emergence. As shown in Fig. 5A, APCP did not change the cytotoxic effect of AG1478. Moreover, as shown in Fig. 5B,C, AG1478 and AG1478+APCP did not alter the percentage of cells expressing active caspase-3 or affect apoptosis/necrosis parameters differently. Likewise, there were no significant differences in Ki-67 expression among the groups (Fig. 5D). AG1478 arrested the cells in the G1 phase compared to controls with a consequent reduction of cells in the S phase. This arrest in the G1 phase was even greater in the combined treatment group results (Fig. 5E).

### AG1478 and AG1478+APCP interfere in GB cells’ motility

**Figure 6:**
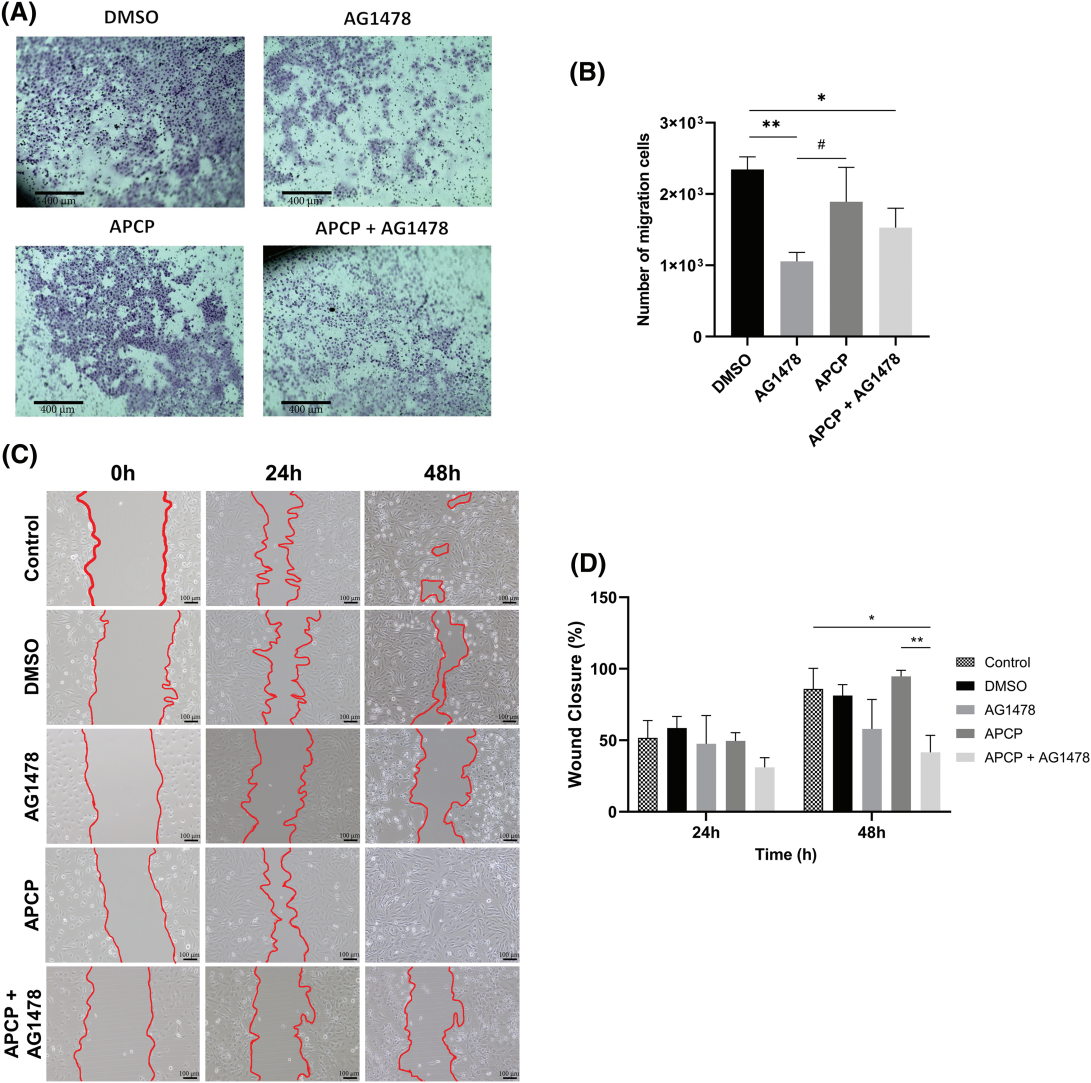
Migration of GB cells after APCP and AG1478 treatments. (A) Representative images of crystal violet staining cells after transwell assay (Scale bar = 400 µm). (B) Quantitative analysis of staining cells following AG1478 and APCP treatments (C) Microscopy image representative of wound closure at 0, 24 and 48 h of exposure to treatments (Scale bar = 100 µm) (D) Wound closure rate at 24 and 48 h of treatments. Data are shown as means ± SD (#*p* < 0.05, *p < 0.05, **p < 0.01).

To explore the effect of the dual inhibition of CD73 and EGFR on cell malignancy, we performed cell migration assays. Considering the transwell assay, AG1478 and AG1478+APCP reduced cell migration after 48 h of treatment (Fig. 6A,B), whereas only AG1478+APCP presented statistically significant changes in the wound healing assay (Fig. 6C,D).

## Discussion

GB is the most prevalent primary brain tumor in adults, characterized by significant heterogeneity and a poor prognosis. Although extensive research has focused on uncovering the intracellular mechanisms driving GB aggressiveness and therapeutic resistance, an effective cure remains elusive. The development of new drugs for GB is hindered by several challenges, including the tumor’s high phenotypic heterogeneity, proliferative and invasive capacity, the presence of glioblastoma stem cells (GSCs), the immunological suppression, the complex interactions between GB cells and the dynamic tumor microenvironment, and acquired resistance [45]. Combining targeted therapies that address multiple cancer drivers may offer an effective strategy to overcoming resistance. Thus, this study aimed to elucidate the effects of combined EGFR and CD73 inhibition in comparison to EGFR monotherapy *in vitro*. Our results revealed elevated EGFR, PD-L1, and CD73 expression levels in the U251-MG cell line. Treatment with AG1478, an EGFR inhibitor, increased resistance markers such as MRP-1, PD-L1, and CD73. On the other hand, the combination of AG1478 with APCP, a CD73 inhibitor, inhibited cell growth through G0/G1 arrest, reduced cell motility, and partially reversed MRP-1 overexpression. To our knowledge, this is the first study to assess the combination of CD73 and EGFR inhibitors in an *in vitro* GB model.

Firstly, we observed considerable variability of EGFR expression in GB cell lines, which correlates with the heterogeneity of this oncogene’s expression in GB patients [46,47]. The activity of EGFR-mediated kinases is critical to promote greater malignancy, making it a promising target to prevent tumor progression [48]. Tyrphostin (AG1478) is a selective EGFR inhibitor that disrupts EGFR autophosphorylation and signaling in cancer, including GB [14,49,50], inhibiting cell migration and invasion [51,52]. Additionally, it has been reported that AG1478 can cause fragmentation of the Golgi apparatus independently of EGFR [53], which is likely related to the morphological cell alterations observed in this study. Moreover, it is possible to observe that the treatment induced the formation of tumor-like tunneling nanotubes (TNTs). TNTs have been described as an adaptative mechanism in resistance development [54], which can be boosted when using tyrosine kinase inhibitors (TKIs) [55].

Although there is a considerable number of studies on EGFR-targeted therapy, consensus has not been reached due to variability in interpatient responses. Areeb et al. demonstrated that TMZ and radiotherapy reduced EGFR expression and that this treatment selected a population of cells with low activity of this receptor, rendering an ineffective response to EGFR-TKIs [56]. Allied with this statement, we did not find an effective response to the combination of TMZ and AG1478 regarding the viability of GB cells. On the other hand, some studies justify the use of simultaneous treatment with TMZ and TKIs [57,58]. The combination of TMZ and nimotuzumab, an anti-EGFR antibody, increased the antitumor response in GB, especially in those patients expressing the EGFRvIII mutation [59]. This treatment combination improved progression-free survival and overall survival compared to TMZ monotherapy [60].

Tumorigenesis involves a complex array of molecular and cellular dysregulations, with chemotherapy resistance frequently emerging in GB, leading to tumor recurrence. Thus, developing treatments that completely eradicate GB cells remains a significant challenge. Our study reflects this issue, as anti-EGFR monotherapy was found to elevate pro-tumor markers, underscoring the limitations of single-agent therapies. The levels of EGFR, immune checkpoints (ICs), and CD73 (and their co-occurrence) are fundamental to leading the GB microenvironment and to an immunosuppressed state. Considering these aspects, we demonstrated that most GB cells present high CD73, PD-L1, and EGFR levels. In addition, inhibition of EGFR by AG1478 upregulated PD-L1 and CD73 expression. Data suggests that EGFR activation can upregulate PD-L1 via different pathways [61–63]. However, EGFR-TKIs resistance development is reported to elevate PD-L1 levels [64,65], which aligns with our study findings. It has been observed that resistance to EGFR inhibitors promotes immune escape by increasing PD-L1 expression in non-small cell lung cancer (NSCLC) [66]. In breast cancer, it was observed that the PD-1/PD-L1 interaction induced the activation of the PI3K/AKT and MAPK/ERK pathways and enhanced MDR1/P-gp expression [67]. In our study, the increase in MRP-1 expression was accompanied by PD-L1 after treatment with AG1478, suggesting that the change in these markers can be established quickly. However, co-treatment with APCP blocked the induction of MRP-1 expression by AG1478, demonstrating a possible mechanism between CD73 and acquired resistance to TKIs.

It is important to note that CD73 expression is very high in relation to the other markers when comparing the MFI and that CD73^+^ cells were also EGFR^+^ and PD-L1^+^. Several studies have demonstrated a strong link between the EGFR and CD73 signaling pathways in various cancers [22,23,68,69]. In this work, we showed that EGFR inhibition increases CD73 expression. Terp and colleagues report that treatment using MAPK inhibitors upregulated CD73 via the compensatory p38 MAPK pathway, reducing the antitumor response [70]. In line with our findings, Griesing et al. have shown that CD73 expression is modulated by the EGFR-ERK signaling pathway [23]. In NSCLC samples with EGFR mutations, CD73 expression is significantly elevated, accompanied by reduced tumor necrosis factor (TNF) expression compared to wild-type tumors [71]. In breast cancer, CD73 has been shown to regulate EGFR expression [72], while Turcotte et al. demonstrated that CD73 contributes to resistance against human epidermal growth factor receptor 2-based targeted therapy [73]. All studies highlighted the potential of targeting CD73 to enhance the efficacy of EGFR therapies.

Thus, we assessed the *in vitro* effects of AG1478 with APCP, a CD73 inhibitor. We showed that the combination of these inhibitors could downregulate the expression of MRP-1. However, since the percentage of cells expressing MRP-1 remained unchanged, it suggests this protein is uniformly downregulated across the entire cell population rather than selectively lost in a subset of cells. In line with this, Torres et al. demonstrated that ADO signalling pathway can influence Akt and ERK1/2 activation, decreasing MRP1 expression in glioblastoma stem-like cells [74]. Inhibiting CD73 reduces MRP1 expression, enhancing the sensitivity of cancer cells to chemotherapy and leading to improved treatment outcomes [75].

Interestingly, we did not notice any differences in the cell proliferation or death of the treated cells. This differed from most studies using the small molecule AG1478 [14,51,76]. Kersting et al. demonstrated that AG1478 increased senescence in Ewing Sarcoma cells [77]. Whereas we demonstrated that the combination of CD73 and EGFR inhibitors suppressed cell growth by inducing G_0_/G_1_ phase cell cycle arrest, which could be induced by p21 and cyclin D1 regulation, as shown by Kim et al. [19]. Carrasco-García and collaborators have also demonstrated that small-molecule EGFR inhibitors (AG1478, gefitinib, and erlotinib) caused G1 arrest in GB cell lines. The arrest promoted, especially by AG1478, was associated with the upregulation of p27 [14]. In addition, Zhang et al. demonstrated that both CD73 silencing and APCP treatment reduced the phosphorylation of Akt, EGFR, and mTOR.

It is worth noting that CD73 also functions as an adhesion molecule, contributing to the migration of both normal and neoplastic cells [78]. It has been shown that CD73 inhibition significantly reduces cell adhesion, migration, and invasiveness in breast cancer, glioma, hepatocellular, and lung carcinoma *in vitro* models [26,79]. Our findings did not bring similar effects on the migratory response of GB cells treated with APCP only, but the combination of CD73 and EGFR inhibition presented reduced GB cell motility. In agreement with our data, Ma et al. demonstrated that AG1478 inhibits the migration and invasion via cell cycle regulation by matrix metalloproteinase-9 [52]. Similarly, Xiao et al. showed that EGFR inhibition diminished cell migration and invasion [80]. Conversely, an anti-CD73 antibody could inhibit breast cancer cell motility by modulating autophagy, whereas EGFR inhibitors induce autophagy [81], promoting drug resistance [82]. However, we did not find changes in autophagy by acridine orange staining (data not shown).

Even though our study offers valuable insights into the effects of combining EGFR and CD73 inhibitors, some limitations should be considered. Our study was conducted using a limited number of cancer cell models, which might not fully represent the heterogeneity in clinical practice. Additionally, long-term studies are essential to evaluate the potential for reducing acquired resistance, such as chronically co-treating cells *in vitro*. The resistance mechanisms between EGFR and CD73 pathways remain poorly understood, as our study focused primarily on the role of CD73 in tumor cells. CD73’s most significant effects may be related to immunomodulation rather than direct tumor cell targeting. Therefore, future investigations should explore the role of this co-treatment *in vivo* and co-culture models with immune cells to understand its potential therapeutic impact better.

## Conclusion

EGFR-targeted therapies can promote resistance in GB. In this sense, we demonstrated that simultaneous inhibition of EGFR and CD73 reverted MRP-1 overexpression induced by anti-EGFR monotherapy, inhibited cell growth by arresting GB cells in the G0/G1 phase and decreased GB cells’ invasiveness. However, the underlying mechanisms still must be elucidated.

## Data Availability

All data generated or analyzed during this study are included in this published article.
